# PMUTs Arrays for Structural Health Monitoring of Bolted-Joints

**DOI:** 10.3390/mi14020311

**Published:** 2023-01-25

**Authors:** Omer M. O. Abdalla, Gianluca Massimino, Fabio Quaglia, Marco Passoni, Alberto Corigliano

**Affiliations:** 1 Department of Civil and Environmental Engineering, Politecnico di Milano, 20133 Milano, Italy; 2 Analog, MEMS & Sensors Group, STMicroelectronics, Via Tolomeo 1, 20010 Cornaredo, Italy; 3 System Research and Applications, STMicroelectronics, Via Paracelso 20, 20864 Agrate Brianza, Italy

**Keywords:** piezoelectric micromachined ultrasonic transducers, ultrasound, multiphysics modelling, SHM applications, acoustoelastic effect

## Abstract

Micro-electro-mechanical systems (MEMS) have enabled new techniques for the miniaturization of sensors suitable for Structural Health Monitoring (SHM) applications. In this study, MEMS-based sensors, specifically Piezoelectric Micromachined Ultrasonic Transducers (PMUT), are used to evaluate and monitor the pre-tensioning of a bolted joint structural system. For bolted joints to function properly, it is essential to maintain a suitable level of pre-tensioning. In this work, an array of PMUTs attached to the head and to the end of a bolt, serve as transmitter and receiver, respectively, in a pitch-catch Ultrasonic Testing (UT) scenario. The primary objective is to detect the Change in Time of Flight (CTOF) of the acoustic wave generated by the PMUT array and propagating along the bolt’s axis between a non-loaded bolt and a bolt in service. To model the pre-tensioning of bolted joints and the transmission of the acoustic wave to and from a group of PMUTs through the bolt, a set of numerical models is created. The CTOF is found to be linearly related to the amount of pre-tensioning. The numerical model is validated through comparisons with the results of a preliminary experimental campaign.

## 1. Introduction

The primary function of bolts in joints is to generate sufficient clamping force to prevent the separation between joint parts. Appropriate pre-tensioning of a bolt makes it less sensitive to fatigue and prevents it from self-loosening when subjected to extreme vibration, stress, or temperature cycles [1]. Both the initial magnitude and the clamping force’s ability to be maintained over time have an effect on the behavior and condition of the bolted joints [2]. As a result, improperly adjusted bolt pre-tensioning can lead to a number of issues in the joints, such as joint separation [1], bolt fatigue failure [3], and nut vibration loosening [4]. Consequently, it is necessary to measure and monitor the pre-tensioning force of the bolts with precision.

Various methods were proposed in the literature to measure the pre-tension of bolts. Strain gauges are a highly accurate method; the use of such technology results in an accuracy of about ± (1–2)% [1,5,6]. They must be installed on a stressed section of the bolt. Therefore, mounting them on an easily accessible end of the bolt is worthless. To take a measurement, there should be easy access to the clamped portion of the bolt shank, which is problematic for many in situ Structural Health Monitoring (SHM) applications. Thus, despite its great level of precision, this approach is best suited for experimental laboratory work. Methods worth mentioning are the Direct Tension Indicators (DTI) [6], the torque control wrench method, the angle control method [7] and Ultrasonic Testing methods (UT) [8]. The DTI are based on measuring the gap between a special washer with bumps on its top surface and the nut. When the gap is smaller than a certain limit, the proper clamping force is assured. This method has an accuracy range of ± (4–12)%. The torque wrench method can only estimate the clamping force from the torque applied to clamp the bolt. However, due to friction loss accompanying the conversion of the torque energy to a clamping force, this method is not accurate [9]. The angle control method uses a linear relationship between the axial force in the elastic region and the angle of rotation; however, it is difficult to find the elastic region in the actual experiment.

The ultrasonic testing method measures the bolt pre-tensioning based on the acoustoelastic effect [10,11,12], which describes the relationship between the velocity of an elastic wave propagating in a solid material and the stress level the material is experiencing. This method adopts a pulse-echo, or a pitch-catch, scenario to measure the Time Of Flight (TOF) needed for an ultrasonic elastic wave to travel along the bolt axis. The TOF measured from an unloaded bolt and a loaded bolt in service are different for two main reasons: (i) bolts in service experience elongation due to the loads exerted on them, hence the elastic wave has a longer path to travel compared to bolts that are unloaded; and (ii) the acoustoelastic effect, which causes the reduction in the elastic wave velocity due to the tensile force the bolt is experiencing along its main axis. Combining the two reasons, a Change in the Time Of Flight (CTOF) is detected in a one-dimensional stress scenario. A linear relationship between the CTOF and the bolt pre-tension is established, leading to a convenient and simple way of measuring and monitoring the status of the bolt loading condition [13,14]. The UT techniques commonly adopted mainly use transducers with bulky Lead Zirconate Titanate (PZT) material for emitting and receiving ultrasonic waves.

This work investigates, through multi-physics modelling and simulation and experimental validation, the use of Piezoelectric Micromachined Ultrasonic Transducers (PMUTs) arrays as a smart and innovative alternative to bulk PZT-based transducers. The main advantages expected through the use of PMUTs are lower cost and reduced volume of PZT, at equivalent performances, when compared to the traditional transducers. In addition, they can be used with embedded electronics and can be integrated into the Internet of Things (IoT) framework.

PMUTs are Micro-Electro-Mechanical Systems (MEMS)-based piezoelectric ultrasonic transducers generating ultrasonic waves that can propagate in air [15,16,17,18,19,20], liquid [21,22,23], or solid media [24]. Piezoelectric materials are used in a wide range of smart micro-systems [25,26]. Unlike bulky piezoelectric transducers, which utilize body modes, PMUTs generate waves through the bending motion of a thin piezoelectric active layer such as PZT [19,27,28,29]. The resonant frequency of the PMUT can be controlled by properly tailoring the in-plane dimension of the flexural membrane and the thicknesses of the constituent layers. As the bandwidth may be narrow for some PMUT designs, it is possible to arrange a set of different diaphragm sizes with different frequencies to broaden the bandwidth and improve the sensitivity of the array [30]. Some micro-fabrication techniques also allow for tuning the acoustic impedance of the PMUTs leading to an enhanced impedance matching with the load media, and therefore having a better acoustic pressure transmission and an improved bandwidth [31]. In addition, they have a reduced voltage requirement, with the ability to be integrated with smart electronic circuits [30]. PMUTs have a wide range of applications such as range finding [32,33], ultrasonic imaging [24,34], and finger-printing recognition [35].

The paper is organized as follows. In Section 2, a model to simulate the propagation of an elastic wave generated from an array of PMUTs inside an unloaded M12 bolt is presented. This model helps understanding the behavior of elastic waves inside a bolted joint in undeformed conditions and to compute the reference TOF for unloaded bolts. A second model is formulated and implemented in Section 3, Section 4 and Section 5 for the study of a bolted joint under static loading conditions i.e., pre-tensioning. Information about the physical elongation of the bolt and the stress history along the axis are extracted. Then, a MATLAB code that utilizes the data gathered from the first and second models is used to implement the acoustoelastic effect. Finally, results from ultrasonic testing of an M12 bolt under different loading conditions are presented. In Section 6, the results of a preliminary experimental campaign on a standard M12 bolt are presented and compared with the results obtained from the numerical models. The last section is devoted to closing remarks.

## 2. Acoustoelastic Effect: Bolt Pre-Tensioning SHM Application

**Figure 1:**
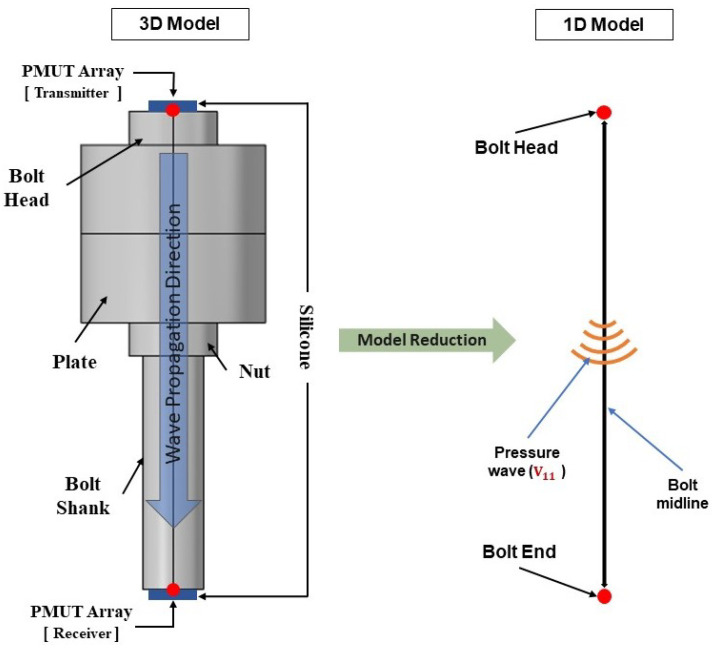
The 3D elastic wave propagation problem can be reduced to a 1D problem based on the fact that both the TOF and CTOF are computed by monitoring only the pressure wave velocity $V_{11}$ which is a function of the static stress $T_{11}$ only, computed along the bolt axis. The bolt axis represents the shortest distance between the mid point on the surface of the bolt head and the bolt end where the arrays are located.

The fastening of a bolt occurs as a result of the tensile stress along the axis of the bolt. The infinitesimal elastic theory fails to express the influence of the state of stress the material is experiencing on the velocity of elastic wave propagation. The acoustoelastic effect explains the dependency of the velocity of an elastic wave in a stressed solid material not only on the second-order elastic constants and density but also on higher-order elastic constants and stress. According to this, the velocity of the wave propagating will change as the stresses changes inside the material. The acoustoelastic effect is also known as the “small on large” theory which considers the propagation of an elastic wave in the material as a small localized state of deformation in addition to the relatively larger deformation caused by the applied stress [36]. Hughes et al. [11,37] were the first to analyze and study the relationship between the stress and the velocity of an elastic wave in an isotropic medium under hydrostatic pressure. Their work was based on the Murnaghan’s theory of finite deformations and third-order elastic constants in the energy. For isotropic materials, three additional constants, *l*, *m*, and *n* are required to describe the material in addition to the Lamé constants λ and μ. Takahashi and Motegi’s work [13,14], derived an equation of motion for an elastic wave in a finitely deformed state using Lagrangian description and the Murnaghan’s finite deformation theory for a unidirectional deformed isotropic solid. Their work shows the need of using the third-order elastic constants in estimating the unknown stresses in structural materials. Considering a longitudinal wave propagating along the same direction as the applied stress, representing a 1D problem, in a uniform isotropic material, a first-order approximation of the velocity relation with the stress can be derived [13]:

(1) $$V_{11}=V_{0}\left(1+\alpha_{11}\frac{T_{11}}{E}\right),$$

in which,

(2) $$V_{0}=\sqrt{\frac{\lambda +2\mu }{\rho }},$$

(3) $$\alpha_{11}=\frac{1}{2(\lambda +2\mu )}\left(5\lambda +10\mu +2l+4m-2v\left(\lambda +2l\right)\right),$$

where ρ is the mass density, λ, and μ are the 2nd-order elastic constants, and *l*, *m*, and *n*, are the 3rd-order elastic constants. For the case of a pre-tensioned bolt, $V_{0}$ and $V_{11}$ represent the stress-independent or zero-load velocity, and stress-dependent acoustic wave propagation velocity in the solid domain, respectively. $T_{11}$ represents the stress along the bolt axis. Keeping track of the behavior of the P-wave, the only source of stresses affecting the wave velocity in the same direction of propagation of the P-wave, i.e., along the bolt axis, is the stress level $T_{11}$. This allows for a reduction of the problem from 3D to 1D, see Figure 1. The relationship presented in Equation (1), which shows the direct and linear relationship between wave velocity and stress, provides a theoretical basis for the axial stress estimation and the assessment of a bolted joint.

The most common available technique adopted in ultrasonic testing is based on measuring the TOF of a traveling acoustic ultrasonic wave either in a pitch catch or a pulse-echo scenario. In the pitch-catch scenario, adopted in this work, the transmitting transducer is usually mounted on top of the bolt head, while the receiving transducer is mounted on the far end of the bolt. The TOF for a pre-tensioned bolt differs from the one of the reference, unloaded, bolt for two reasons.

1. The bolt stretches as it is tightened, thus the path length of the acoustic wave increases.
2. The reduction in the acoustic wave velocity due to the acoustoelastic effect.

For a bolt loaded in an elastic regime, both changes are linear functions of the level of tension the bolt is experiencing. Hence, the CTOF is also a linear function of the bolt pre-tensioning [1].

The TOF in the pre-tensioned bolt, in a pitch-catch scenario, is defined as follows [38]:

(4) $$TOF=\int_{0}^{L}\frac{\left(1+\varepsilon (z)\right)}{V_{11}\left(z\right)}dz,$$

where *z* is the direction along the bolt axis, *L* is the length of the bolt, ε(z) is the strain along the bolt axis, and $V_{11}$(z) is the stress-dependent acoustic wave velocity given in Equation (1). Based on linear elasticity, the following relation holds:

(5) $$T_{11}\left(z\right)=E\varepsilon \left(z\right).$$

Substituting Equations (1) and (5) into Equation (4), the TOF can be expressed as:

(6) $$\begin{array}{cc} \mathrm{TOF} & =\int_{0}^{L}\frac{\left(1+\varepsilon (z)\right)}{V_{0}\left(1+\alpha_{11}\frac{T_{11}\left(z\right)}{E}\right)}dz\;=\;\frac{1}{V_{0}}\int_{0}^{L}\frac{\left(1+\varepsilon (z)\right)}{\left(1+\varepsilon \left(z\right)\alpha_{11}\right)}dz. \end{array}$$

By assuming that $(\varepsilon \left(z\right)\;\alpha_{11})\ll 1$, using the Maclaurin expansion of $1/\left(1+\varepsilon \left(z\right)\alpha_{11}\right)\approx 1-\left(\varepsilon \left(z\right)\alpha_{11}\right))+{\left(\varepsilon \left(z\right)\alpha_{11}\right)}^{2}-{\left(\varepsilon \left(z\right)\alpha_{11}\right)}^{3}$, and neglecting the higher order terms, the following expression can be obtained:

(7) $$\begin{array}{cc} \mathrm{TOF} & =\frac{1}{V_{0}}\int_{0}^{L}\left(1+\varepsilon \left(z\right)\right)\left(1-\varepsilon \left(z\right)\;\alpha_{11}\right)\;dz\\ \; & =\frac{1}{V_{0}}\int_{0}^{L}\left(1+\varepsilon \left(z\right)-\varepsilon \left(z\right)\;\alpha_{11}-\varepsilon {\left(z\right)}^{2}\;\alpha_{11}\right)\;dz. \end{array}$$

By neglecting the term $\varepsilon {\left(z\right)}^{2}\;\alpha_{11}$ and integrating:

(8) $$\begin{array}{cc} \mathrm{TOF} & =\frac{1}{V_{0}}\;\left(L_{0}+\delta -\alpha_{11}\;\delta \right)\\ \; & =TOF_{0}+\frac{1}{V_{0}}\;\left(1-\alpha_{11}\right)\;\delta , \end{array}$$

having defined

(9) $$\begin{array}{c} \delta =\int_{0}^{L}\varepsilon \left(z\right)dz,\;TOF_{0}=\frac{L_{0}}{V_{0}}.\; \end{array}$$

δ is the elongation of the bolt due to pre-tensioning, while $TOF_{0}$ is the time of flight before pre-tensioning. The CTOF can therefore be computed as:

(10) $$\begin{array}{c} CTOF=TOF-TOF_{0}=\frac{\delta }{V_{0}}\;\left(1-\alpha_{11}\right) \end{array}$$

For a bolt loaded elastically, and reduced to a 1D problem, the value of δ is defined as:

(11) $$\delta =\frac{P}{K}$$

where *P* is the pre-tensioning force and *K* is the equivalent stiffness of the pre-tensioned bolt, equal to EA/*L* in which *A* is an equivalent cross-sectional area and *L* is the deformed length. Equations (10) and (11), show that the CTOF is linearly proportional to the bolt pre-tensioning. To develop this relationship, a set of numerical models are needed. The first model simulates the pre-tensioning of a bolted joint under several loading conditions. For different levels of pre-tensioning forces *P*, the axial stress $T_{11}$(*z*) along the bolt axis and the elongation δ can be evaluated. A second model is created to simulate a pitch-catch UT scenario using an array of PMUTs for transmission and reception. From this model, the value of the Reference Time of Flight ($TOF_{0}$) can be evaluated. Finally, a MATLAB code is needed to combine the acoustoelastic effect with the results obtained from the numerical models.

## 3. Pre-Tensioned Bolt Modeling

**Figure 2:**
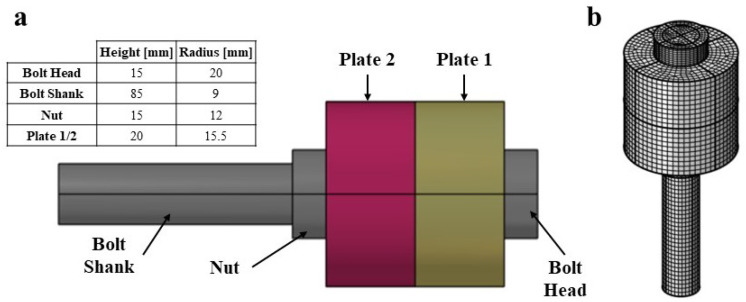
(**a**) Model of the M12 Bolt. (**b**) Meshed model.

**Figure 3:**
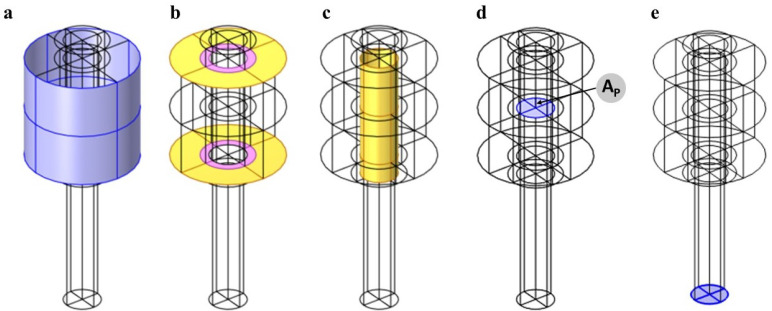
M12 bolt model Boundary Conditions (BC). (**a**) Fixed BC on the exterior surface of the plates. (**b**) Contact BC, where the contact problem is solved for, at the bolt head/plate and nut/plate shared boundaries. (**c**) Contact BC at the threaded region of the plate holes/thread and nut/thread. (**d**) Pre-tensioning load application surface. (**e**) Spring foundation BC.

An M12 steel bolt is chosen and the FE code COMSOL Multiphysics V5.5 (COMSOL, Inc. Stockholm, Sweden) is used to create and analyze the bolted joint system. The FE model consists of the following elements: the bolt, made of a bolt-head and a bolt-shank, a nut, and a clamped element represented by the plates; see Figure 2. For the sake of simplicity, the threaded part of the bolt shank extending along the bolt length is not depicted. Additionally, the hexagonal shape of the bolt head is replaced by a cylinder with a diameter equal to the standard nominal diameter of an M12 bolt. The cylinder representing the bolt head has a height of 7.5 mm and a radius of 10 mm while the bolt shaft has a height of 100 mm and a radius of 6.85 mm. The length of the clamped region, represented by the height of the two plates, is equal to 40mm. The numerical modeling of a bolted-joint under pre-tensioning is carried out under the following hypotheses: material and geometrical nonlinearities, implementation of hyperelastic Murnaghan’s constitutive model, contact conditions between surfaces of the clamped elements with the upper surface of the nut and the lower surfaces of the head of the bolt, and contact conditions between the bolt-shank and the hole in the clamped elements; see Figure 3. All structural elements are modeled using structural steel having Young’s modulus and Poisson’s ratio of 200 GPa and 0.3, respectively, and with values of the 2nd and 3rd elastic constants equal to (λ = 1.15 × 10${}^{11}$, μ = 7.69 × 10${}^{10}$, l = −3.0 × 10${}^{11}$, m = −6.2 × 10${}^{11}$, n = −7.2 × 10${}^{11}$) Pa [14,39]. The system of bolted joint, shown in Figure 2, is entirely meshed using quadratic wedge elements. The mesh size is selected with reference to the dimensions of the discretized bolt portion; for example, the bolt head mesh is generated by meshing to the top surface of the bolt head with an element size equal to the radius of the bolt head divided 8. The surface mesh is then swept along the height of the bolt head creating wedge elements. A similar meshing process is adopted for the bolt shank, the nut and the plates.

To apply the prescribed pre-tensioning, a surface boundary A${}_{P}$, see Figure 3, is created in the bolt-shank perpendicular to the bolt axis at the level the two clamped plates are meeting. A pre-tensioning load is applied on A${}_{P}$ in the form of pre-tensioning stress $\sigma_{P}$. This method ensures that the prescribed pre-tensioning is attained inside the bolt. The exterior boundary of the plates is modeled as fixed boundaries. This model involves a high number of contact surfaces, such as the contact problem at the bolt head/plate, nut/plate, and the plate and nut hole/thread interaction surfaces. A fine-scale computation of the bolted joint assemblies, in which the geometry of the threaded regions is modeled, is too computationally expensive. Hence, more simplified computational methods for accurately solving contact problems may be necessary to handle such computationally demanding problems. A non-penetration condition is enforced at the contacting boundaries. Both contributions of the normal and friction tractions are modeled at the contacting surface. A friction coefficient of 0.1 is used. Additional treatment is devoted to the contacting surfaces in the threaded regions of the plates and nut threads and bolt threads. For the threaded region, the actual geometry of the threads is handled by a mathematical formulation of the contact condition. Hence, some additional geometrical design parameters can be numerically implemented at the mating threads. A 30${}^{\circ }$ half-angle of a single thread is used with a lead length of 1.25 mm. Adopting this simplified modeling technique of the pre-tensioned bolt is computationally very efficient and provides accurate results [40]. A spring foundation BC is enforced on the bottom end of the shank, primarily to reduce instability, which is a common concern in numerical modeling of contact problems. To solve the contact problem at the contacting surfaces, the Augmented Lagrangian technique is adopted. The technique is implemented through the algorithmic Uzawa’s Method, which provides an iterative update of the contact tractions at the contacting surface [41]. The non-linear contact problem of the pre-tensioning of bolted joints is numerically solved by adopting the double dogleg method, which is highly suitable for non-linearly constrained contact problems. The method is based on a combination of the Steepest descent and Newton-Raphson methods, which guarantee a good convergence of the solution.

**Figure 4:**
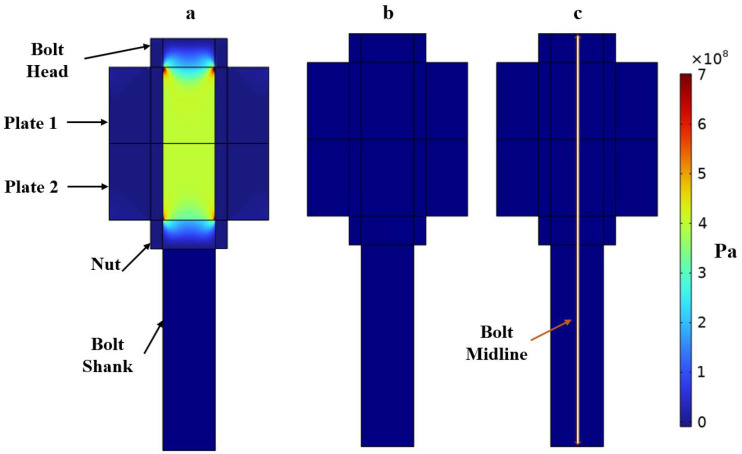
Stress distribution on a cross-section of the bolted joint under a pre-tensioning level of 450 MPa. (**a**) $T_{11}$, (**b**) $T_{12}$ and (**c**) $T_{13}$. It can be noticed that the shear stress $T_{12}$ and $T_{13}$ are by far much smaller and negligible with respect to $T_{11}$.

**Figure 5:**
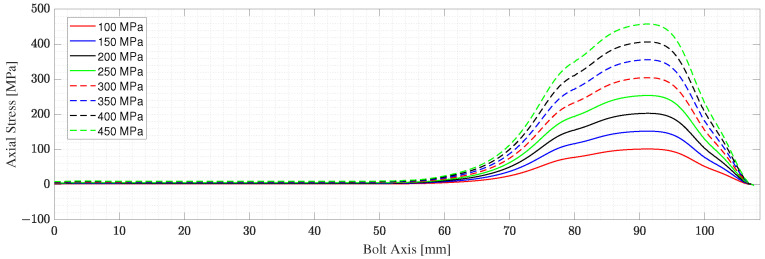
Stresses ($T_{11}$) along the bolt axis for different pre-tensioning load.

**Figure 6:**
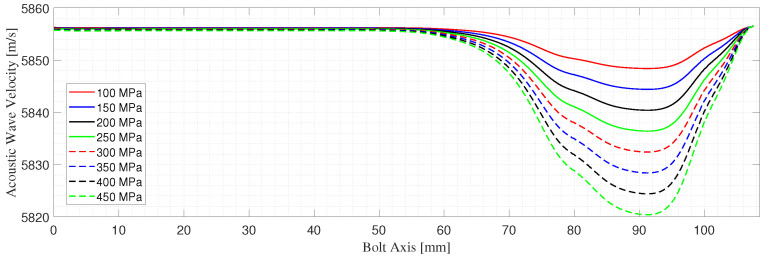
Acoustic wave velocity ($V_{11}$) variation along the bolt axis for different pre-tensioning load.

The bolt is studied under seven different loading conditions ranging from 100 to 450 MPa with steps of 50 MPa. The loading cases considered are within the elastic range. The results extracted from this model are the stress along the bolt axis and the deformation of the bolt for each loading case. The total deformation presented in Table 1 is obtained by integrating the strains along the bolt axis. Figure 4 shows the $T_{11}$ stress distribution for two of the loading cases simulated, namely, the case of a pretension level of 200 and 400 MPa. However, only the variations of $T_{11}$ along the bolt axis are of interest for the particular application presented in this work, since the problem is reduced to 1D; see Figure 1. The stresses along the bolt axis $T_{11}$(z) for different pre-tensioning forces are shown in Figure 5. The bolt shank stress is observed to differ from zero by negligible amounts. These forces result from the spring foundation BC assigned to the end surface of the shank. It is possible to adjust the spring foundation’s stiffness to minimize the effect on the bolt shank while preserving the solution’s stability. From the stress $T_{11}$(z), the stress-dependent acoustic wave velocity $V_{11}$(z) is computed along the length of the bolt using Equation (1). The acoustoelastic, or small-on-large, theory is based on the assumption that the time-dependent deformation due to the wave propagation is much smaller than the static state of deformation due to the applied stress, which indeed holds for this work [36]. This makes the mapping of the velocity on the deformed length, as presented in Figure 6, of the bolt, admissible.

## 4. PMUT Arrays for SHM of Bolted Joints

**Figure 7:**
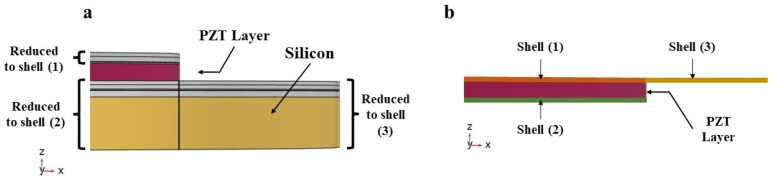
Detailed representation of the PMUT radius showing: (**a**) the multi-layers of a PMUT radius; (**b**) the Reduced Order Model (ROM) of the PMUT [22].

**Figure 8:**
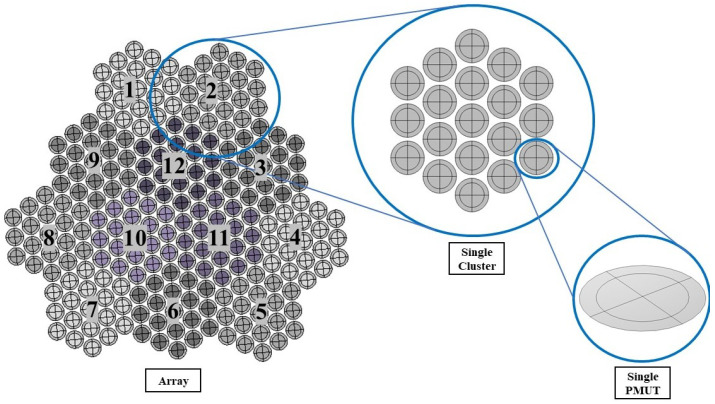
Configuration of the array of PMUTs [22].

Smart designing and miniaturizing of sensors have led to the realization of transducers that have low power consumption with high accuracy and efficiency, which made them suitable to be used in smart, wireless, and highly reliable real-time monitoring and communication applications. Various SHM applications rely on transducers that utilize bulky PZT materials to emit and detect acoustic waves. Replacing traditional portable transducers with smart and miniaturized transducers for SHM, such as PMUT arrays, will provide many advantages such as improving the accuracy and sensitivity in addition to the possibility of smart integration with on-chip embedded systems and IoT frameworks. A single PMUT, which are diaphragm-like shaped multi-layered systems, has an overall thickness of 7.445 μm. The active PZT layer has a thickness of 2 μm and a radius of 69 μm. The structural layer made of silicon has a thickness of 4 μm with a radius of 94 μm. The multi-layered system of each PMUT is modeled using shell elements, except for the active PZT layer, see Figure 7, thus leading to a great reduction of the number of Degrees of Freedom (DOF) and of the computational burden [22]. The PMUT array adopted in this work consists of 228 individual PMUTs, in which every 19 PMUTs is electronically connected in parallel, creating a cluster. A total of 12 electrically independent clusters arranged uniquely generate an array, see Figure 8. The PMUTs cavities are considered to be fully released with a total thickness of 400 μm. This eliminates any possibility of squeeze-film damping from the backside of the PMUTs. Hence, the array of PMUTs that is modeled and simulated for this application is a reduced-order model that considers only the vibrating plates. Details about the modeling, simulation and sensitivity analysis of this array have been discussed in our previous work [22].

**Figure 9:**
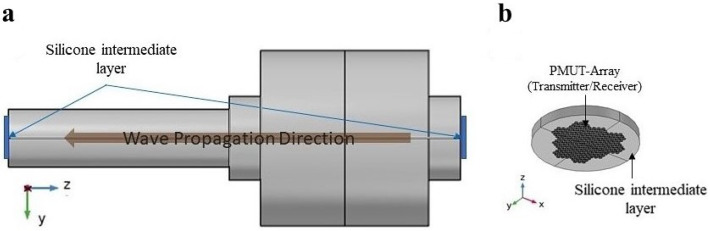
Pitch-Catch ultrasonic testing technique. (**a**) Direction of the pressure acoustic wave propagation along the bolts access emitted from the Transmitting PMUT array and detected by the receiving PMUT array. (**b**) PMUT array deposited on an intermediate silicone rubber layer.

**Figure 10:**
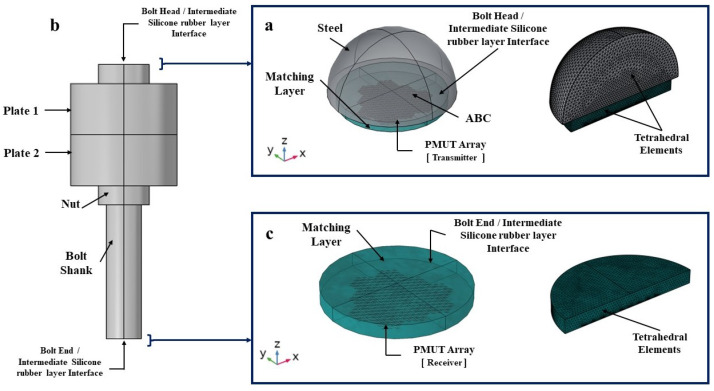
(**a**) Modeling of the transmission phase from the PMUT array into a truncated solid domain at the bolt head. (**b**) Modeling of the bolt only to simulate the acoustic wave propagation generated from the PMUT array. (**c**) Modeling of the reception phase of the acoustic wave arriving at the receiving array of PMUTs.

The wave generated from the transmitting PMUT will propagate along the bolt until it reaches the end of the bolt where the PMUT receiver is placed, see Figure 9. The receiver will read a voltage generated as a consequence of the mechanical vibrations in the PZT active layers in the PMUTs produced by the acoustic wave. The difference between the time of transmission and reception defines the TOF. A very high computational burden is faced when simulating this model. Starting from modeling two large arrays of PMUTs attached to the huge solid domain of the bolt, having the same elastic material properties adopted in the pre-tensioned bolt model, the number of DOF solved for it will be very high. Hence, the modeling and simulation of this problem are divided into three different phases, governed by three models, as described here below; see Figure 10.

**Figure 11:**
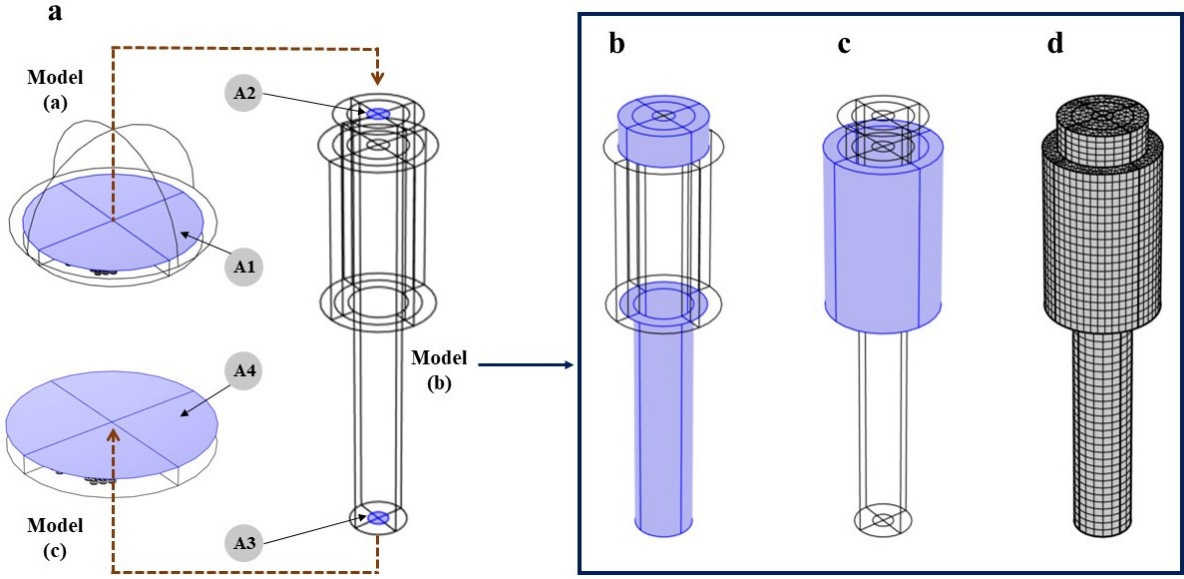
(**a**) The bolt head and end/silicone layer interfaces in the three models. (**b**) Free BC. (**c**) Absorbing boundary layer added as an additional exterior layer surrounding the plates structure to simulate an infinite domain with respect to the acoustic minimum wave length ($\lambda_{s}$) in solid domain. (**d**) Bolted joint meshed using quadratic wedge elements for the elastic wave propagation simulation.

**Figure 12:**
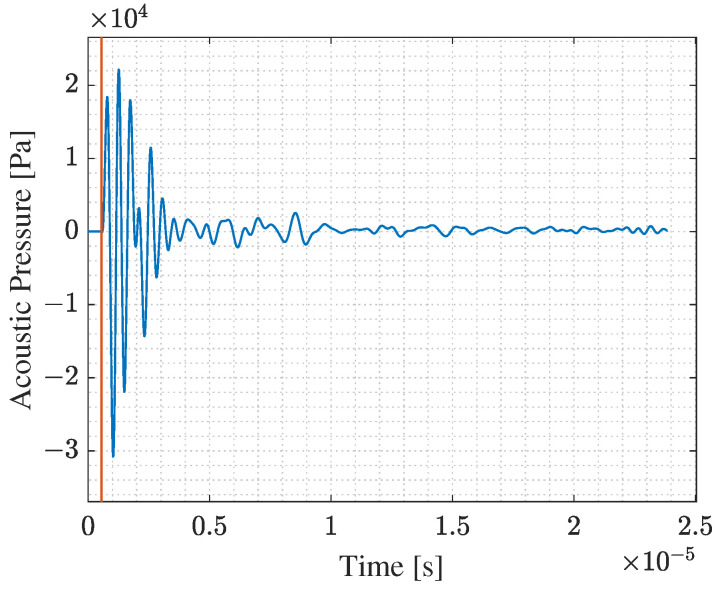
Computed acoustic pressure history at bolt head/silicone layer interface.

**Figure 13:**
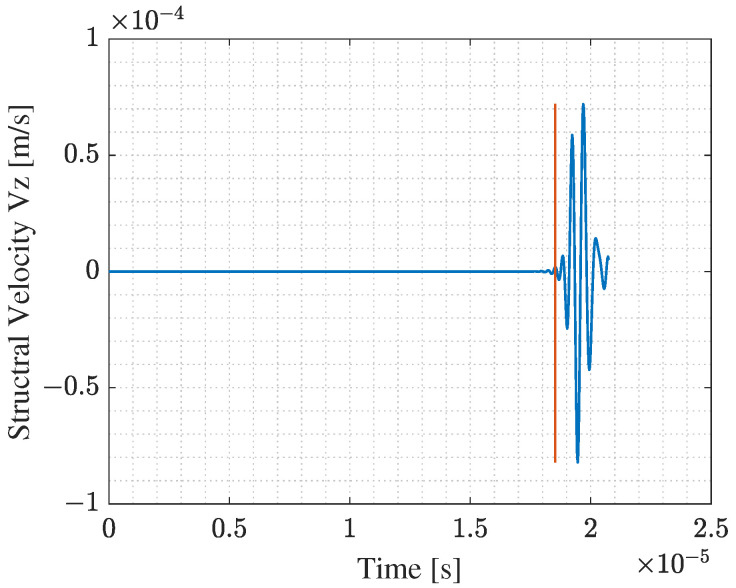
Computed structural velocity (Vz) history at bolt end/silicone layer interface.

- Model (a) simulates the transmission phase of an acoustic wave emitted from a PMUT array into the intermediate silicone rubber layer and a truncated part of the bolt head solid domain. The array is deposited on top of a cylindrical silicone layer, having 0.5 mm thickness and 2.5 mm radius, which is a rubber-like material. The array and the silicone layer are attached to the truncated solid domain, which is represented by a hemisphere with a radius of 2.875 mm. All PMUTs in the array are activated using a single-cycle sinusoidal input signal with an amplitude of 5 V and a central frequency of 1.81 MHz. This frequency corresponds to the fundamental frequency of the PMUT in contact with the silicone layer. The acoustic pressure at the bolt head/silicone layer interface A1, see Figure 11, is computed at every shared mesh node of the interface between the two domains, and stored.
- In model (b), the acoustic pressure history computed and stored from the model (a), is assigned as a boundary load at a surface area A2, which is equivalent to A1, at the bolt head. This model simulates the acoustic wave propagation into the solid bolt domain only. Figure 12 shows the acoustic pressure history that represents the output of the model (a) and the input for the model (b). The acoustic pressure history arriving at the bolt end is then computed (and stored) at the surface A3 at the bolt end, which corresponds to the bolt end/silicone layer interface.
- Similarly, in model (c), the acoustic pressure computed at surface A3 is assigned as a boundary load at the top surface of the silicone layer A4. Figure 13 shows the acoustic wave velocity history that represents the output of the model (b) and the input for the model (c). This model simulates the reception phase of the acoustic wave arriving at the PMUT array in receiving mode.

**Figure 14:**
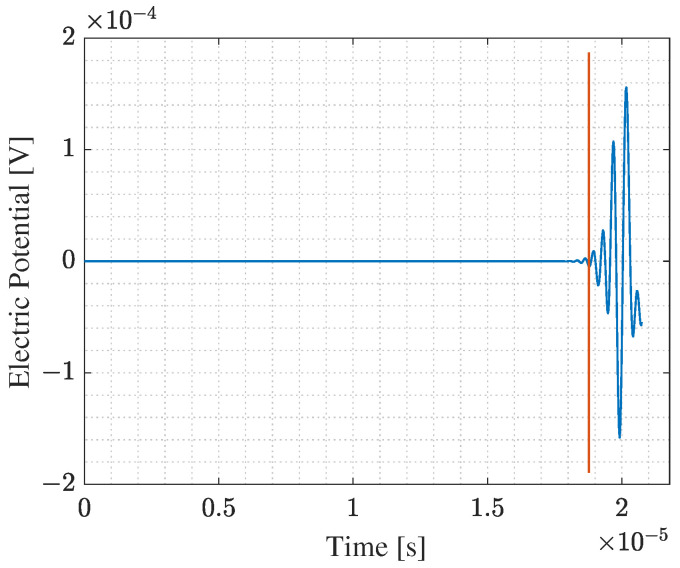
Electric potential gain computed from the central PMUT of the central cluster 12 of the receiving PMUT array at the end of the bolt.

In Figure 10, the exterior surface of the hemisphere representing the truncated domain of the bolt head is assigned with an Absorbing Boundary Condition (ABC). This BC is added to simulate an infinite domain, with respect to the minimum wavelength ($\lambda_{m}$) of the acoustic wave in steel, by allowing an almost negligible reflection of an incident wave. At the bolt head/silicone layer interface, an Acoustic-Structure Interaction (ASI) condition is enforced. In the silicone layer (acoustic domain), the pressure equation is solved, while in the steel domain (solid domain), the equation of motion is solved. The equations governing this study are discussed thoroughly in a previous work [22]. The multiphysics simulation is solved in the time domain utilizing the implicit generalized alpha time integration scheme with an adopted integration step of 1/(64 × $f_{0}$), where $f_{0}$ is the central frequency, 1.81 MHz, at which PMUTs are excited. The simulation of the transient elastic wave propagation in the large solid domain of the bolt is solved with a Discontinuous Galerkin Method (DG-FEM) that uses the time-explicit Runge-Kutta of the 4th order time integration scheme. For all the cases, the acoustic domain is meshed using quadratic tetrahedral elements having a size of 8.18 × 10${}^{-4}$ m, 1/10 of the minimum wavelength ($\lambda_{a}$) of the acoustic wave, while the solid domain is meshed, using quadratic tetrahedral elements for the hemisphere and quadratic wedge elements for the bolt solid domain, with a size of 1.61 × 10${}^{-4}$ m, corresponding to 1/20 ($\lambda_{s}$). Activating the PMUT array at the bolt head using a single cycle sinusoidal input signal with an amplitude of 5 V, with a central frequency of 1.81 MHz leads to a generation of acoustic pressure of 30 kPa at the bolt head/silicone layer interface. The propagating wave reaches the bolt end/silicone layer interface with enough energy that can give an electric potential gain at the receiving PMUT of an amplitude of more than 2 mV Peak-to-Peak; see Figure 14. The model allows for the computation of the value of $TOF_{0}$, needed for the calculation of the value of CTOF for the bolt under pretension, of the level of pressure transmitted to the bolt head and received at the bolt end and of the electric potential gained at the receiving PMUT array. The computed values of the acoustic pressure and of the electric potential support the use of PMUT arrays for SHM of bolted joints.

The reference value of $TOF_{0}$ needed to compute the CTOF can be computed from Figure 12 and Figure 13, considering the difference between the time at which the first evident peak of the acoustic pressure shows, indicated by the red line shown in Figure 12, and the time at which the first evident peak of the structural velocity shows in Figure 13. The obtained value is equal to 1.8357 × 10${}^{-5}$ s. The length of the bolt is equal to 107.5 mm, while the elastic wave propagation velocity in the steel bolt is 5856.4 m/s. Hence, the theoretical value of $TOF_{0}$ is 1.83559 × 10${}^{-5}$ s. Comparing the computed and the theoretical value of $TOF_{0}$, the numerical model simulates accurately the elastic wave propagation problem. Additionally, Figure 14, shows that the electric potential amplitude gained at the receiving PMUT array is enough to sense the TOF. The slight time shift of the red line shown in Figure 14, from the one in Figure 13, is equal to 3.3752 × 10${}^{-7}$ s, which is equivalent to the time needed for an acoustic pressure wave to travel through the thickness of the silicone layer. Hence, the models show high accuracy in simulating the emission, propagation, and reception phases of the acoustic wave.

## 5. Implementation of the Acoustoelastic Effect

The acoustoelastic effect is implemented in a MATLAB code following the hyperelastic Murnaghan’s constitutive model. For each pre-tensioning scenario, the CTOF is obtained following the steps below.

1. The deformed length $L_{f}$ is divided into (N) segments of equal length denoted by Δx.
2. Starting from the stress history along the bolt axis $T_{11}$(z), shown in Figure 5, the stress-dependent velocity of the elastic wave in each point along the bolt axis is obtained using Equation (1). The values of the 2nd and 3rd elastic constants used to obtain the coefficient $\alpha_{11}$ are (λ = 1.15 × 10${}^{11}$, μ = 7.69 × 10${}^{10}$, l = −3.0 × 10${}^{11}$, m = −6.2 × 10${}^{11}$, n = −7.2 × 10${}^{11}$) Pa [14,39].
3. The average velocity in each segment is obtained as an average velocity of the two nodal velocity values of the segment. For a generic (i-th) segment:
  (12) $$V_{i,avg}=\frac{V_{i}+V_{i+1}}{2},$$
  with *i* having values between 1 and N + 1.
4. The time needed for the acoustic wave with an average velocity $V_{i,avg}$ to travel through an element of the length Δx is given as:
  (13) $$t_{i}=\frac{\Delta x}{V_{i,avg}}.$$
5. The TOF is simply the sum of the N values of $t_{i}$:
  (14) $$TOF=\sum_{i=1}^{N}t_{i}.$$
6. Finally, the CTOF is given by:
  (15) $$CTOF=TOF-TOF_{0}.$$

Since this numerical model is implemented on the deformed length L${}_{f}$, the obtained CTOF contains the contributions to the change in the time of flight due to both the physical elongation and the acoustic wave velocity reduction, i.e., the acoustoelastic effect.

Table 2 contains, for each pre-tensioning scenario, the value of the TOF computed using the above procedure. A linear relationship between the CTOF and the corresponding level of the bolt pre-tensioning is obtained.

## 6. Experimental Validation

**Figure 15:**
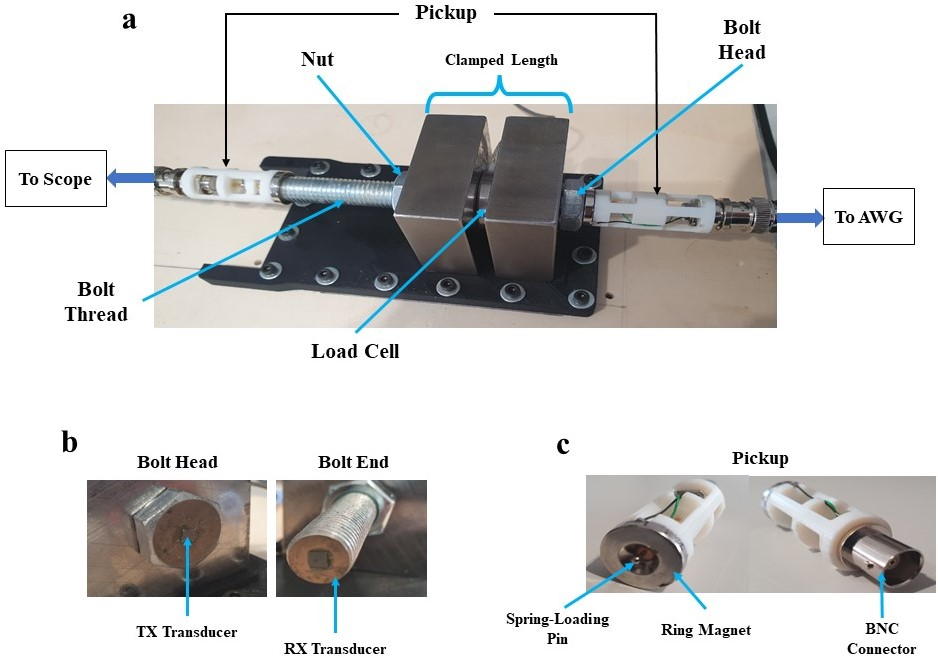
(**a**) Experimental setup of the ultrasonic testing of a bolted joint. (**b**) Transmitter and receiver transducers attached to bolt head and end, respectively. (**c**) The pickup unit with BNC and spring-loading pin connectors.

This section presents the results of a preliminary experimental campaign on the use of UT for an M12 bolted joint. The objective is to measure the CTOF of an ultrasonic acoustic wave propagating inside a clamped bolt. Figure 15 demonstrates the experimental setup used for ultrasonic testing of an M12 bolted joint. Both the head and the end surfaces of the bolt are reworked using an Electrical Discharge Machining (EDM) process to decrease the surface roughness and enhance the adhesion of the transducers to the bolt. The clamped length of the threaded region of the bolt shaft is 47.5 mm. The transducers used for transmission and reception are the UTensor II from MicroControl, which are 3 mm in length × 3 mm in width × 0.1 mm in height piezoelectric ceramics. They are adhered to the bolt surface using Loctite 680. The substitution of the Silicone intermediate material with Loctite 680 will have two levels of impact. TOF is expected to change because Loctite has a different characteristic value of wave velocity propagation than Silicone. However, this will not affect the primary sought-after results of CTOF. The second consideration is the impact of the mismatch in acoustic impedance between Loctite 680 and the steel solid domain. Depending on the acoustic impedance mismatch, this could result in a higher or lower acoustic pressure being transmitted into the bolt. The transmitting transducer is excited with an impulsive input at a central frequency of 7.5 MHz with a 2 V peak-to-peak amplitude from an Arbitrary Waveform Generator (AWG). A magnetic pickup unit is mounted on top of the transducer with a cable connecting it to the ultrasonic measurement unit. The experiment is done in a pitch-catch UT scenario with the transmitter located at the bolt head and the receiver at the bolt end. The setup allows for real-time monitoring of the CTOF of the ultrasonic wave propagating in the bolted joint during the tightening process. The experimental campaign is performed at this stage solely to measure the CTOF with respect to the variation of the axial stress of the bolt. The results of the experiments are not dependent on the use of piezoelectric ceramics or the rest of the equipment. They only provide the experimental data with which the numerical results are compared and validated on the basis of the acoustoelastic effect in steel bolts.

**Figure 16:**
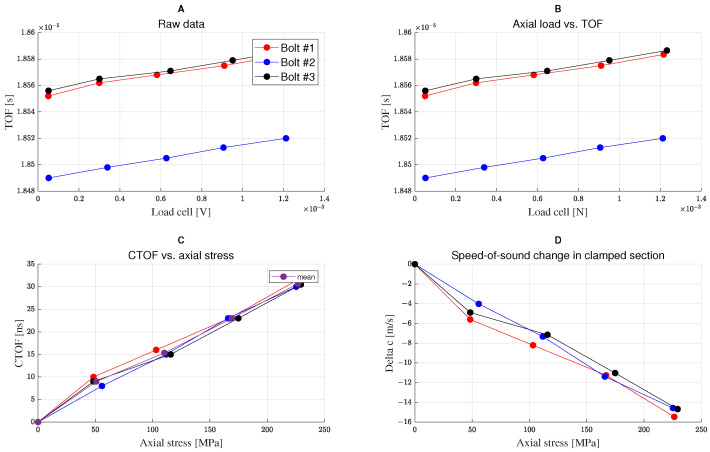
(**A**) Raw data measured from the load cell. (**B**) TOF of the ultrasonic wave vs bolt axial load measured by the load cell. (**C**) A linear relationship between the CTOF and the “pre-tensioning” bolt axial stress. The three data sets’ mean value curve are represented. (**D**) A linear relationship between the change in the ultrasonic wave propagation velocity and the “pre-tensioning” bolt axial stress.

**Figure 17:**
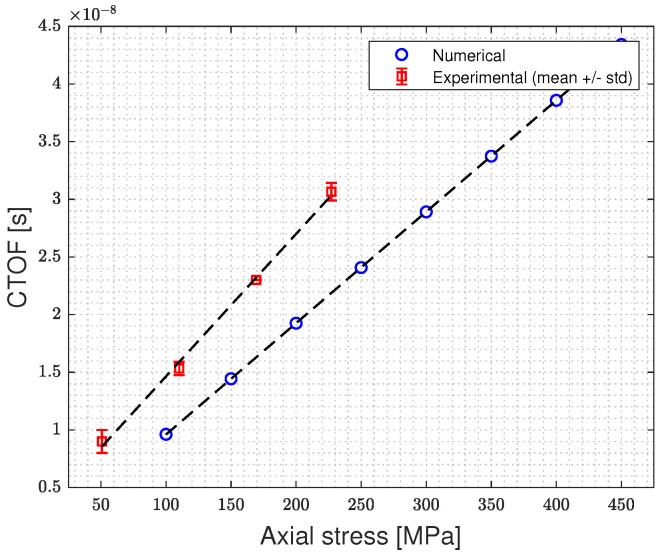
Numerical and experimental linear relationship between the CTOF of the ultrasonic wave and the bolt axial stress “pre-tensioning”.

Figure 16 illustrates the outcomes of the experimental campaign performed on three different M12 bolts: the TOF is plotted versus the load cell signals, the CTOF and the wave propagation velocity are plotted against the pre-tensioning axial stress. The difference in TOF measurements between bolts is due to the varying thickness of the intermediate layer utilized in each measurement. This, however, has no impact on the CTOF measurement. For comparison with the numerical results, the mean value curve of the CTOF vs. bolt pre-tensioning derived from the three bolt measurements is chosen, see Figure 17. Both experimental and numerical results predicted a level of CTOF ranging between 100–450 µs for the investigated pre-tensioning loading range. The differences between the numerical and experimental results are due to the uncertainty in the characteristic values of the 3rd-order elastic constants (l, m, n) of the bolt used in the experimental campaign.

## 7. Closing Remarks

Bolted joints are critical structural elements that support the functionality of many structural systems, such as large tanks, containers, bridges, and complex mechanical systems, such as wind turbines and moving vehicles. Monitoring and assessment of the bolt pre-tensioning are crucial for the integrity of the bolted joints.

In this work, a MEMS-based UT technique was investigated for its suitability of the real-time monitoring of bolt pre-tensioning. The work aimed at investigating the possibility of the replacement of bulk-PZT-based transducers with PMUT arrays.

A pitch-catch UT technique was used to measure the bolt pre-tensioning. The TOF which the ultrasonic wave needs to propagate along the pre-tensioned bolt axis is linearly proportional to the level of pre-tensioning. The TOF increases with the pre-tensioning due to the physical elongation of the bolt and to the acoustoelastic effect. Both reasons are linearly proportional to the pre-tensioning force.

A set of numerical models was created to reproduce this linear relationship. A model was created to study the behavior of an M12 bolted joint system under different loading conditions. Two arrays, each consisting of 228 PMUTs, were modeled to transmit and receive acoustic waves in a pitch-catch UT scenario, with the transmitting array located at the bolt head and the receiving array at the bolt end.

The transmitting array was found to be able to emit a pressure level of 30 kPa into the bolt, using a single cycle sinusoidal input signal with an amplitude of 5 V, that reaches the far end with enough energy that can generate an electric potential gain, at the receiving transducer, of an amplitude of more than 2 mV Peak-to-Peak.

The values of the acoustic pressure transmitted and received and the electric potential gained by the PMUT array indicate the suitability of using PMUT arrays for SHM applications of bolted joints. The applicability is not restricted to M12 bolts only. Higher density arrays are anticipated to generate greater pressure intensities that propagate over greater distances before losing energy and becoming difficult to detect. Consequently, inspection of bigger bolt sizes may be possible.

The numerical simulations of the transmission and repetition phases of the acoustic wave allowed for an accurate estimation of the reference TOF${}_{0}$. The hyperelastic constitutive model proposed by Murnaghan was implemented in a MATLAB code and a linear relationship was obtained between the CTOF of the ultrasonic wave and the level of pre-tensioning in the bolt.

A preliminary experimental UT was conducted on a clamped M12 Bolt. The numerical and experimental results were found to be in good agreement.

Future work will involve experimental tests to precisely measure the characteristic values of the elastic constants of the steel material and an extensive campaign with the application of PMUT arrays.

## Figures and Tables

**Table 1:** Bolt axis elongation (δ) computed using Equation (9), for different values of pre-tensioning.

Pretension [MPa]	Bolt Axis Elongation (δ) [mm]
100	0.01906
150	0.02858
200	0.03809
250	0.04760
300	0.05710
350	0.06659
400	0.07608
450	0.08556

**Table 2:** Computed TOF for the different bolt pre-tensioning values.

Pretension [MPa]	TOF [s]
100	$1.836560\times 10^{-5}$
150	$1.837042\times 10^{-5}$
200	$1.837524\times 10^{-5}$
250	$1.838006\times 10^{-5}$
300	$1.838489\times 10^{-5}$
350	$1.838973\times 10^{-5}$
400	$1.839457\times 10^{-5}$
450	$1.839941\times 10^{-5}$

## Data Availability

Not applicable.

## Abbreviations

The following abbreviations are used in this manuscript:

ABC	Absorbing Boundary Condition
ASI	Acoustic-Structure Interaction
AWG	Arbitrary Waveform Generator
BC	Boundary Conditions
CTOF	Change in the Time of Flight
DG-FEM	Discontinuous Galerkin Method
DOF	Degrees of Freedom
DTI	Direct Tension Indicator
EDM	Electrical Discharge Machining
IoT	Internet of Things
MEMS	Micro-Electro-Mechanical Systems
PARDISO	PARallel DIrect SOlver
PMUTs	Piezoelectric Micromachined Ultrasonic Transducers
PZT	Lead Zirconate Titanate
ROM	Reduced Order Model
SHM	Structural Health Monitoring
TOF	Time of Flight
TOF${}_{0}$	Reference Time of Flight
UT	Ultrasonic Testing
